# Posttraumatic stress disorder (PTSD) prevalence: an umbrella review

**DOI:** 10.1017/S0033291724002319

**Published:** 2024-11

**Authors:** Alexa Schincariol, Graziella Orrù, Henry Otgaar, Giuseppe Sartori, Cristina Scarpazza

**Affiliations:** 1 Department of General Psychology, University of Padova, Padova, Italy; 2 Padova Neuroscience Center (PNC), University of Padova, Padova, Italy; 3 Department of Neuroscience, University of Padova, Padova, Italy; 4 Department of Surgical, Medical and Molecular Pathology and Critical Care Medicine, University of Pisa, 56126 Pisa, Italy; 5 Faculty of Law and Criminology, KU Leuven, Leuven, Belgium; 6 Faculty of Psychology and Neuroscience, Maastricht University, Maastricht, the Netherlands; 7 IRCCS S. Camillo Hospital, Venezia, Italy

**Keywords:** assessment, intentional event, non-intentional event, posttraumatic stress disorder, prevalence, PTSD, self-report, structured clinical interview, traumatic event, umbrella review

## Abstract

Posttraumatic stress disorder (PTSD) is one of the most serious and incapacitating mental diseases that can result from trauma exposure. The exact prevalence of this disorder is not known as the literature provides very different results, ranging from 2.5% to 74%. The aim of this umbrella review is to provide an estimation of PTSD prevalence and to clarify whether the prevalence depends on the assessment methods applied (structured interview *v*. self-report questionnaire) and on the nature of the traumatic event (interpersonal *v*. not-interpersonal). A systematic search of major databases and additional sources (Google Scholar, EBSCO, Web of Science, PubMed, Galileo Discovery) was conducted. Fifty-nine reviews met the criteria of this umbrella review. Overall PTSD prevalence was 23.95% (95% confidence interval 95% CI 20.74–27.15), with no publication bias or significant small-study effects, but a high level of heterogeneity between meta-analyses. Sensitivities analyses revealed that these results do not change after removing meta-analysis also including data from underage participants (23.03%, 95% CI 18.58–27.48), nor after excluding meta-analysis of low quality (24.26%, 95% CI 20.46–28.06). Regarding the impact of diagnostic instruments on PTSD prevalence, the results revealed a lack of significant differences in PTSD prevalence when structured *v*. self-report instruments were applied (*p* = 0.0835). Finally, PTSD prevalence did not differ following event of intentional (25.42%, 95% CI 19.76–31.09) or not intentional (22.48%, 95% CI 17.22–27.73) nature (*p* = 0.4598). The present umbrella review establishes a robust foundation for future research and provides valuable insights on PTSD prevalence.

## Introduction

Posttraumatic stress disorder (PTSD) is the most serious and incapacitating mental disease that can result from trauma exposure. Traumatic events including natural disasters, accidents, sexual violence, and child abuse are common all over the world, and their mental health consequences, such as PTSD, are equally widespread. According to estimates, people experience on average about three traumatic events during their lifetime (Kessler et al., 2017).

Although the majority of people who experience traumatic situations recover spontaneously and exhibit a normal pattern of resilience, a significant proportion of those who experience trauma do encounter psychological repercussions, such as acute stress disorder, difficult bereavement, adjustment disorder, and depression. Among these, PTSD is one of the most common. On the one hand, the lifetime worldwide prevalence of PTSD in the general population is around 5.6% (Koenen et al., 2017). On the other, PTSD point prevalence varies widely, even when regarding a traumatic event of the same nature. For instance, the meta-analysis conducted in 2019 by Wang et al. (2019) on the development of PTSD after being exposed to hurricanes and typhoons yielded a prevalence of 17.81% (95% confidence interval [CI] 12.63–23.67), with data ranging from 1% in the study by Rubens, Vernberg, Felix, and Canino (2013) to a peak prevalence of 62% in the study conducted by Guo et al. (2016). This variability can also be observed when comparing meta-analyses concerning the same traumatic event (e.g. Dai et al., 2016; Sepahvand, Mokhtari Hashtjini, Salesi, Sahraei, & Pirzad Jahromi, 2019 reported a PTSD prevalence after earthquakes of 23.66% and 58%, respectively) and increases further when considering traumatic events of different nature (e.g. 5.02% PTSD prevalence following pregnancy and birth in Yildiz, Ayers, & Phillips, 2017 *v*. 47% PTSD prevalence following war in Sepahvand et al., 2019).

Numerous psychological and economical pre- and post-traumatic factors have been proven to raise the likelihood of developing and maintaining PTSD, including personality traits, prior mental health conditions (Perrin et al., 2014), female sex (Kessler et al., 2005; Perrin et al., 2014), country of origin and sociodemographic variables (Koenen et al., 2017), and specific changes in gene expression (Kessler et al., 2005; Perrin et al., 2014). The presence of multiple moderating factors influencing PTSD prevalence explains the wide gap in results between studies.

After experiencing a traumatic incident, it is common to endure some psychological distress and PTSD-related symptoms (Sayed, Iacoviello, & Charney, 2015). However, many individuals with PTSD-related symptoms will see the majority or the totality of those symptoms completely disappear within a month, displaying trajectories of resilience and reflecting a path of natural recovery (Littleton, Axsom, & Grills-Taquechel, 2011); in other cases, symptoms will fluctuate throughout time, including remission and re-emergence (Feder et al., 2016; Galatzer-Levy & Bryant, 2013).

The World Mental Health Surveys (https://www.hcp.med.harvard.edu/wmh/) of the World Health Organization (World Health Organization, 2022) indicate that between 25% and 40% of PTSD-diagnosed people will recover within 12 months, with many of those cases resolving within the first 6 months (Koenen et al., 2017). According to meta-analytic statistics (Morina, Wicherts, Lobbrecht, & Priebe, 2014; Steinert, Hofmann, Leichsenring, & Kruse, 2015), however, nearly 50% of PTSD sufferers will have a chronic condition, especially if the mental illness is not treated.

Despite the relevance of the disorder, there is still considerable confusion and debate surrounding its diagnosis. PTSD is diagnosed using criteria established by two primary systems: the Diagnostic and Statistical Manual of Mental Disorders (DSM) and the International Classification of Diseases (ICD). The DSM, published by the American Psychiatric Association, is commonly used in the United States and many other countries. The latest edition, DSM-5-TR (American Psychiatric Association, 2022), categorizes PTSD under *Trauma- and Stressor-Related Disorders*. It requires exposure to trauma through direct experience, witnessing, learning about a traumatic event involving a close associate, or repeated exposures to aversive details of such events. PTSD diagnosis in the DSM-5-TR includes four symptom clusters: intrusion, avoidance, negative alterations in cognitions and mood, and alterations in arousal and reactivity. Symptoms must persist for more than one month and significantly impair functioning. On the other hand, the ICD, published by the World Health Organization, is widely used globally, particularly in Europe. The latest edition, ICD-11 (World Health Organization, 2022), classifies PTSD within *Mental, Behavioral, and Neurodevelopmental Disorders*. It emphasizes exposure to extremely threatening or horrifying events and identifies three core symptoms: re-experiencing the traumatic event, avoidance of trauma-related thoughts and situations, and a persistent perception of heightened current threat. Symptoms should last for several weeks and cause significant distress or impairment in important areas of functioning.

While both systems have similar criteria, the DSM-5-TR includes a broader range of symptoms, especially related to cognition and mood, whereas the ICD-11 focuses on fewer core symptoms, emphasizing the perception of current threat. Moreover, a different strategy guided the revision process of the two manuals: on the one hand, the experts selected to review the DSM-IV were required to provide a strong empirical basis for each adjustment of the diagnostic criteria; on the other hand, the working group responsible for the publication of the ICD-11 based its decisions solely on a consensus among experts. Finally, the ICD-11 maintained the three symptom clusters from the DSM-4 and introduced two ‘sibling disorders’: PTSD and complex PTSD. The ICD-11 approach is debated by Friedman, Vermetten, and their respective research groups (Friedman, Schnurr, & Keane, 2021; Vermetten, Baker, Jetly, & McFarlane, 2016) because it excludes from the diagnostic criteria a number of symptoms that are not specific to PTSD, such as insomnia, irritability, difficulty concentrating, and social withdrawal. According to the authors, this would be inconsistent with the categorization of symptoms of other mental disorders and problematic because it could result in a deprivation of diagnosis to symptomatic individuals.

A second factor that creates significant impediment to reaching consensus between professionals consists in the choice of assessment methodology. There is now widespread agreement that diagnosing PTSD is a challenging endeavor that requires careful consideration of the person's presenting complaints, co-occurring psychological and physical issues, occupational and social functioning, as well as cultural and other contextual variables that may be associated with the presentation and progression of PTSD symptomatology (Friedman et al., 2021). As a result, a variety of methods for assessing PTSD have been developed, including structured diagnostic interviews conducted by a clinician, self-report psychological exams and questionnaires, and psychophysiological measurements. Structured and semi-structured diagnostic interviews are both common and recommended practices in research settings, but their use in clinical settings is less widespread (Keane, Buckley, & Miller, 2003). In general terms, this may be due to the specialized training required to conduct these interviews properly, as well as time or financial restrictions (Friedman et al., 2021). Self-report assessments are typically more affordable and less time consuming than structured interviews (Friedman et al., 2021). They can be especially helpful when conducting PTSD screenings or when used in conjunction with structured interviews to provide physicians more information and monitor treatment outcomes over time. Nevertheless, to diagnose PTSD, self-report measures should not be employed in isolation since they lack the validity and reliability of structured clinical interviews (Jablensky, 2002). Due to biases in answers, misunderstandings of the patient filling in the questionnaire, and contextual variables, any self-report measure has the potential to cause significant inaccuracy (Jablensky, 2002).

PTSD examination is further complicated when considering the features of the traumatic event that resulted in the disorder. Some research suggested that a person's likelihood for developing PTSD depends on the nature of stressful incident they experience (Santiago et al., 2013). Compared to other types of traumatic event exposures, sexual assault and other interpersonal trauma have been shown to have more severe and debilitating psychological effects (Breslau, 2009; Pietrzak, Goldstein, Southwick, & Grant, 2011). Particularly, Santiago et al. (2013) have revealed that traumatic experiences seen as non-intentional (e.g., natural disasters) are less likely to cause long-lasting symptoms of PTSD than intentional ones (e.g., assault, rape, torture, etc.). However, no studies have been conducted to date to investigate predictors or risk factors that may regulate the various trajectories of PTSD in people exposed to intentional and non-intentional exposures.

Considering the many challenges involved in assessing PTSD and the effect these have on the number of diagnoses, we conducted an umbrella review (Fusar-Poli & Radua, 2018; Ioannidis, 2009) to provide an estimation of the prevalence of the disorder following various types of traumatic events. More specifically, we performed an in-depth analysis to evaluate the variability in the prevalence of PTSD depending on the assessment method and the nature of the traumatic event (interpersonal *v*. not-interpersonal).

## Method

The current umbrella review was carried out adopting the Preferred Reporting Items for Systematic Reviews and Meta-Analyses (PRISMA) guidelines (Page et al., 2021) and the Joanna Briggs Institute methodology for umbrella reviews (Aromataris et al., 2014). The PRISMA flowchart (Haddaway, Page, Pritchard, & McGuinness, 2022) was used to represent the screening phase and the selection process. The study protocol was pre-registered with the International Prospective Register of Systematic Reviews (PROSPERO; CRD42022322800).

### Search strategy

Google Scholar, EBSCO (CINAHL Complete, Psychology and Behavioral Sciences Collection, APA PsycInfo, APA PsycArticles), Web of Science, PubMed, and Galileo Discovery were searched of observational studies investigating PTSD prevalence. For each database, titles, abstracts, subject headings, and general keywords were searched with no language or time constraints. The literature search began on the 17th of March 2022, and all databases and additional sources were searched from inception until the 3rd of April 2022. Moreover, further studies were found by means of the ‘related articles’ function provided by ConnectedPapers (https://www.connectedpapers.com/) and by tracing the references from review articles and the identified papers. If two or more meta-analyses included a complete or substantial overlap in primary studies, the most recent or broader one was employed (see online Supplementary material S1 for the search strategy).

### Inclusion and exclusion criteria

Studies were included in the umbrella review if they met the following inclusion criteria:

- Meta-analysis of individual observational studies (case-control, cohort, cross-sectional, longitudinal and ecological studies) assessing PTSD prevalence;
- studies considering any established diagnosis of PTSD defined by the ICD or the DSM;
- studies reporting PTSD prevalence after traumatic events;
- studies reporting sufficient data for the analyses (e.g. number of PTSD diagnoses among people exposed to the traumatic event and number of individuals who experienced the traumatic event or PTSD prevalence).

Exclusion criteria were the following:

- Meta-analysis that did not present study-level data with 95% CIs;
- systematic reviews with no quantitative analysis;
- reviews that incorporated theoretical studies or published opinion as their primary source of evidence.

**Figure 1.**
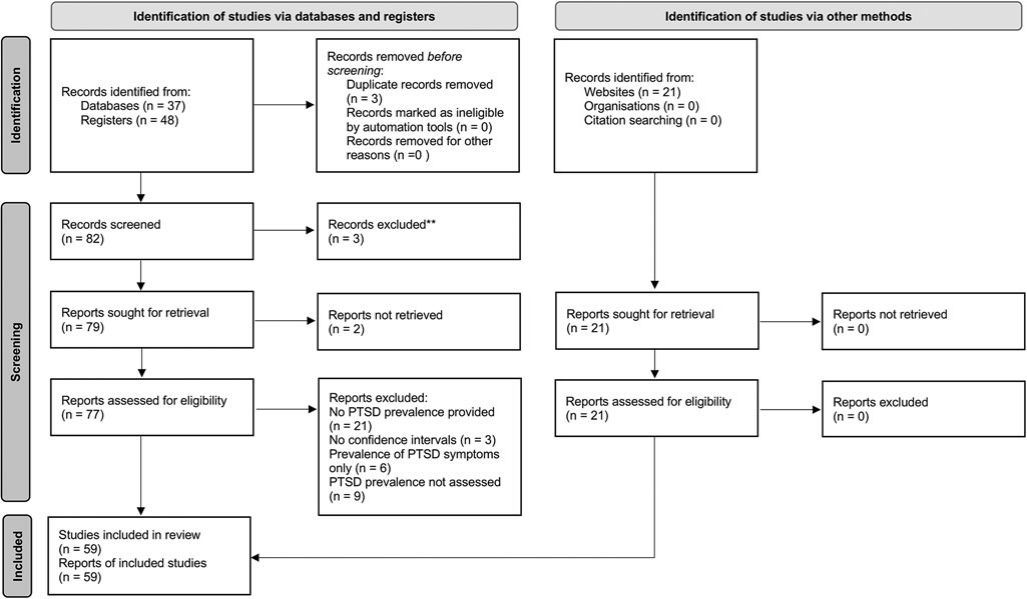
PRISMA flowchart of literature search.

See Fig. 1 for the PRISMA flow diagram of study screening and selection.

### Data extraction and selection

A systematic approach was used for data extraction. Each meta-analysis was checked to ensure that it met the eligibility criteria. The following factors were then extracted and entered in an Excel table: first author and year of publication; type of traumatic event; PTSD assessment method; type of study; target population (adults, adolescents, or children); number of cases and total sample size; PTSD prevalence and corresponding 95% CI; heterogeneity; and *p*-value. The data extracted from the meta-analyses are reported in Table 1.

**Table 1.** Characteristics of the meta-analyses included in the umbrella review

Author(s) and year of publication	Context	Assessment method	Type of study	Age	*k*	Events	Sample	Prev. (%)	95% CI	*I*^2^ (%)	*p*	AMSTAR-2 index	Type of event
Abbey et al. (2015)	Cancer	Interview + self-report	Cross-sectional + longitudinal	>18	7	528	4189	12.6	7.4–20.7	79.2	<0.01	Moderate	Not-interpersonal
Agbaria et al. (2021)	Political violence – children	Interview + self-report	Cross-sectional + longitudinal	<18	25	5443	15 121	36	30–41	98.6	<0.001	Moderate	Interpersonal
Alisic et al. (2014)	Interpersonal trauma – children	Interview	Cross-sectional + longitudinal	<18	42	566	3563	15.9	11.5–21.5	NA	NA	Moderate	Both
Al-Saadi et al. (2022)	Cancer – children	Interview + self-report	Cross-sectional + longitudinal	<18	9	158	755	20.9	13.28–29.73	83.5	<0.001	Low	Not-interpersonal
Amiri (2022)	Immigrants	Interview + self-report	Cross-sectional + longitudinal	>14	51	10 310	41 240	25	22–29	99.47	<0.001	Moderate	Unknown
Arora et al. (2022)	COVID-19	Interview + self-report	Cross-sectional	>18	6	1302	3945	33	0–86	99.7	<0.001	High	Not-interpersonal
Ayano et al. (2021)	Homeless people	Interview + self-report	Cross-sectional	>18	19	5576	20 364	27.38	21.95–33.57	97.67	<0.001	Moderate	Unknown
Badenes-Ribera et al. (2021)	Homicide	Interview + self-report	Cross-sectional	>14	11	321	754	42.6	38.0–47.4	19.5	0.405	Low	Interpersonal
Baranyi, Cassidy, Fazel, Priebe, and Mundt (2018)	Prison	Interview + self-report	Cross-sectional	>18	50	2880	21 099	13.65	10.4–17.3	93.5	NA	High	Unknown
Blackmore et al. (2020)	Refugees – children and adolesc.	Interview	Cross-sectional	<18	7	155	681	22.71	12.79–32.64	91.1	0	High	Unknown
Blackmore et al. (2020)	Refugees	Interview	Cross-sectional	>18	22	1459	4639	31.46	24.43–38.50	97.2	0	High	Unknown
Burgess et al. (2021)	Parents following pediatric medical events	Interview + self-report	Cross-sectional	>18	45	2043	6743	30.3	25.3–35.5	93.57	>0.001	Low	Interpersonal
Cabizuca, Marques-Portella, Mendlowicz, Coutinho, and Figueira (2009)	Parents of children with chronic illnesses	Interview + self-report	Cross-sectional + longitudinal	>18	10	421	1845	22.8	16.4–29	NA	NA	Low	Interpersonal
Cénat, McIntee, and Blais-Rochette (2020)	Earthquake	Interview + self-report	Cross-sectional + longitudinal	>6	24	2274	7997	28.44	17.68–42.37	99.31	NA	Critically low	Not-interpersonal
Cénat et al. (2021)	COVID-19	Self-report	Cross-sectional	>18	13	6680	30 449	21.94	9.37–43.31	99.85	NA	Moderate	Not-interpersonal
Chen and Liu (2015)	Floods	Interview + self-report	Cross-sectional	>6	14	6390	40 600	15.74	11.25–20.82	98.3	<0.0001	Moderate	Not-interpersonal
Cohen et al. (2010)	War (military service)	Self-report	Cross-sectional + longitudinal	>18	20	39 082	417 985	9.35	5.8–12.9	NA	NA	Critically low	Interpersonal
Cruz, White, Bell, and Coventry (2020)	Extreme weather events (UK)	Self-report	Cross-sectional	>18	4	413	1359	30.36	11.68–49.05	99	<0.01	Moderate	Not-interpersonal
Dai et al. (2016)	Earthquake	Interview + self-report	Cross-sectional + longitudinal	>6	46	18 005	76 101	23.66	19.34–28.27	99.5	<0.001	High	Not-interpersonal
Dai et al. (2018)	Road traffic accidents – children and adolesc.	Interview + self-report	Cross-sectional + longitudinal	<18	11	306	1532	19.95	13.63–27.09	90	<0.01	Moderate	Not-interpersonal
DiMaggio and Galea (2006)^a^	Terrorism	Interview + self-report	Cross-sectional	>18	42	NA	NA	15.9	0.6–35.9	NA	NA	Critically low	Interpersonal
Dworkin (2020)^a^	Sexual violence	Interview	Cross-sectional	>18	21	NA	NA	36.2	31–41	96.8	<0.01	Moderate	Interpersonal
Edmondson et al. (2012)	Acute coronary syndrome	Interview + self-report	Cross-sectional + longitudinal	>18	24	286	2383	12	9.0–16.0	80.85	<0.001	Low	Not-interpersonal
Edmondson et al. (2013)	Stroke and transient ischemic attack	Interview + self-report	Cross-sectional	>18	9	148	1138	13	11.0–16.0	89.49	<0.001	Moderate	Not-interpersonal
Fulton et al. (2015)	War (freedom operations)	Interview + self-report	Cross-sectional + longitudinal	>18	33	114 250	494 589	23.1	20–26	NA	NA	Low	Interpersonal
Gualtieri, Ferretti, Masti, Pozza, and Coluccia (2020)	Prisoners’ offspring	Interview + self-report	Cross-sectional + longitudinal	>6	6	377	2512	15	0.81–24.9	92.637	<0.001	Moderate	Unknown
Henkelmann et al. (2020)	Refugees	Interview + self-report	Cross-sectional + longitudinal	>6	59	3853	13 288	29	23–36	99.2	<0.001	High	Unknown
Hines et al. (2014)^a^	War (Iraq and Afghanistan)	Interview + self-report	Cross-sectional + longitudinal	>18	55	NA	NA	10.13	8.06–12.20	99.4	<0.001	Low	Interpersonal
Hoell et al. (2021)	Refugees	Interview + self-report	Cross-sectional	>6	25	3589	12 002	29.9	20.8–38.7	NA	NA	High	Unknown
Hoppen and Morina (2019)	War (global pop.)	Interview	Cross-sectional	>18	30	3901	16 383	23.81	19.54–28.35	NA	NA	Critically low	Interpersonal
Hoppen, Priebe, Vetter, and Morina (2021)	War (global pop.)	Interview + self-report	Cross-sectional	>18	22	4088	15 420	26.51	22.17–31.10	98	<0.001	High	Interpersonal
Hosseinnejad et al. (2022)	Earthquake	Interview + self-report	Cross-sectional + longitudinal	>6	16	4292	7719	55.6	49.9–61.3	96	0	Critically low	Not-interpersonal
Liang, Zeng, Liu, Xu, and Liu (2021)	Earthquake – elderly	Unknown	Cross-sectional	>18	10	1208	4834	25	20–29	91.9	0.001	Moderate	Not-interpersonal
Lin et al. (2018)	Road traffic accidents	Interview + self-report	Cross-sectional + longitudinal	>6	15	1514	6804	22.25	16.71–28.33	97.1	<0.001	High	Not-interpersonal
Loignon et al. (2020)^a^	Traumatic brain injury (TBI)-military and civilian pop.	Interview + self-report	Cross-sectional + longitudinal	>18	31	NA	NA	27.1	21.8–33.1	94.2	NA	Moderate	Unknown
Morina, Stam, Pollet, and Priebe (2018)	War (civilians)	Interview	Cross-sectional	>18	30	4910	18 886	26	0.23–0.31	97	<0.001	High	Interpersonal
Musanabaganwa et al. (2020)	Genocide	Interview + self-report	Cross-sectional	>7	19	2937	11 746	25	16–36	99.5	0	Moderate	Interpersonal
Nagarajan, Krishnamoorthy, Basavarachar, and Dakshinamoorthy (2022)	COVID-19	Interview + self-report	Cross-sectional	>18	13	175	1093	16	9.0–23	87.9	<0.001	High	Interpersonal
Nguyen, Guajardo, Sahle, Renzaho, and Slewa-Younan (2022)	Refugees	Interview + self-report	Cross-sectional + longitudinal	>18	5	341	1101	31	22–41	95.26	NA	High	Unknown
Rezayat et al. (2020)	Earthquakes and floods – children and adolesc.	Interview + self-report	Cross-sectional + longitudinal	>6	39	11 212	58 396	19.2	18.6–19.7	NA	NA	Critically low	Not-interpersonal
Rodrigues, Barletta, and Nery (2021)	Traumatic events of various kinds (not interp.)	Interview + self-report	Cross-sectional + longitudinal	>6	26	4508	24 276	18.57	13.8–23.87	96.22	<0.0001	Low	Not-interpersonal
Rona et al. (2016)	War (UK service personnel)	Self-report	Cross-sectional + Longitudinal	>18	8	85	3405	2.5	1.6–3.4	59.2	0.086	Critically low	Interpersonal
Sahebi et al. (2021)	Health care workers during COVID-19 pandemic	Interview + self-report	Cross-sectional	>18	7	43 732	323 459	13.52	9.06–17.98	65.5	0.008	Moderate	Not-interpersonal
Sepahvand et al. (2019)	Childbirth	Interview + self-report	Cross-sectional	>18	7	632	2527	25	14–37	97.82	NA	High	Interpersonal
Sepahvand et al. (2019)	Job (emergency staff)	Interview + self-report	Cross-sectional	>18	6	348	1161	30	4.0–66	99.35	NA	High	Interpersonal
Sepahvand et al. (2019)	Earthquake	Interview + self-report	Cross-sectional	>18	9	1980	3414	58	41–75	99.08	NA	High	Not-interpersonal
Sepahvand et al. (2019)	War	Interview + self-report	Cross-sectional	>6	9	3314	7052	47	32–63	98.84	NA	High	Interpersonal
Sepahvand et al. (2019)	Burn	Interview + self-report	Cross-sectional + longitudinal	>18	2	53	133	40	27–66	NA	NA	High	Not-interpersonal
Sepahvand et al. (2019)	Accidents	Interview + self-report	Cross-sectional	>6	2	86	779	11	5.0–21	NA	NA	High	Not-interpersonal
Sepahvand et al. (2019)	Sexual violence	Interview + self-report	Cross-sectional	>18	2	148	200	74	67–80	NA	NA	High	Interpersonal
Siqveland et al. (2017)	Chronic pain	Interview + self-report	Cross-sectional	>18	21	655	6750	9.7	5.2–17.1	98.6	NA	High	Not-interpersonal
Souza et al. (2011)	Peacekeepers	Interview + self-report	Cross-sectional	>18	12	731	13 782	5.3	3.4–7.2	96.8	NA	Critically low	Interpersonal
Steel et al. (2009)	War (torture and other traumatic events)	Interview + self-report	Cross-sectional	>18	145	19 696	64 332	30.6	26.3–35.2	97.6	<0.001	Low	Interpersonal
Stein et al. (2013)	War (human rights abuses – civilians)	Interview + self-report	Cross-sectional	>18	118	12 458	40 188	31	27–35	98.64	<0.0001	High	Interpersonal
Suomi, Bolton, and Pasalich (2023)	Parents in child protection services	Interview + self-report	Cross-sectional	>16	11	1805	7848	23	17.0–29.0	97	0	Critically low	Interpersonal
Swartzman et al. (2017)	Cancer	Interview + self-report	Cross-sectional + longitudinal	>18	76	1565	16 755	9.34	4.96–20.2	NA	NA	Moderate	Not-interpersonal
Van Praag et al. (2019)^a^	TBI in civilian populations	Interview + self-report	Cross-sectional + longitudinal	>18	31	NA	NA	15.64	12.88–18.40	82	<0.00001	High	Not-interpersonal
Wang et al. (2019)	Typhoon or hurricane	Interview + self-report	Cross-sectional + longitudinal	>6	39	7680	43 123	17.81	12.63–23.67	99.6	<0.001	Moderate	Not-interpersonal
Warmerdam et al. (2019)	Parents of children with cancer	Interview + self-report	Cross-sectional + longitudinal	>18	31	2408	9262	26	22–32	96	NA	Low	Interpersonal
Wilcoxon (2019)	Child trauma effect on parents (doctoral thesis)	Interview + self-report	Cross-sectional	>18	41	743	4370	17	14.1–20.0	83.71	<0.001	High	Interpersonal
Woolgar et al. (2022)	Various types of traumas – children	Interview	Cross-sectional + longitudinal	<7	18	417	1941	21.5	13.8–30.4	94.9	NA	High	Both
Wu, Wang, Cofie, Kaminga, and Liu (2016)	Cancer	Interview + self-report	Cross-sectional + longitudinal	>18	34	1543	16 076	9.6	7.9–11.5	91.1	<0.001	Moderate	Not-interpersonal
Yildiz et al. (2017)	Pregnancy and birth	Interview + self-report	Cross-sectional + longitudinal	>18	59	1218	24 267	5.02	3.52–7.12	NA	NA	Moderate	Interpersonal
Yuan et al. (2021)	Infectious disease pandemics in the 21st century	Interview + self-report	Cross-sectional + longitudinal	>6	73	46 066	203 831	22.6	19.9–25.4	99.7	0	Moderate	Interpersonal
Yunitri et al. (2022)	COVID-19	Interview + self-report	Cross-sectional + longitudinal	>18	63	21 892	124 952	17.52	13.89–21.86	NA	NA	High	Interpersonal

*k*, number of studies included in the meta-analysis; events, number of PTSD diagnoses among people exposed to the traumatic event; sample, number of people exposed to the traumatic event; CI, 95% confidence interval; *I*^2^, heterogeneity; prev., prevalence; *p*, *p*-value; probab., probability; NA, not applicable.

a No sample size provided.

AMSTAR-2 (A Measurement Tool to Assess Systematic Reviews; Shea et al., 2017), a 16-point evaluation tool assessing the methodological quality of systematic reviews and meta-analysis, was used to evaluate the quality of the included meta-analyses (for the quality assessment, see online Supplementary material S3). Test–retest reliability, content validity, and inter-rater agreement are all strong points of AMSTAR-2. The following categories served as the foundation for evaluating reviews: (a) formulation of the research question; (b) provision of an a priori design; (c) justification of the study designs of the included studies; (d) a thorough review of the literature; (e) study selection; (f) data extraction; (g) a list of excluded studies, as well as an explanation of why they were excluded; (h) thorough description of the key features of the included studies; (i) risk of bias assessment; (j) details regarding the funding sources; (k) techniques for statistically combining results; (l) assessment of the potential impact of individual study bias risk on the meta-analysis result; (m) discussion/interpretation of the potential impact of individual study bias risk on the meta-analysis result; (n) discussion of the heterogeneity seen in the study results; (o) probability of publication bias; and (p) conflict of interest disclosure for the study's authors. Seven of these 16 domains, referred to as ‘critical domains’, can have a significant impact on the validity of the assessment and its result (domains *b*, *d*, *g*, *i*, *k*, *m*, and *o*). There are three possible responses for each item: a full yes, a partial yes, or a no.

Although AMSTAR-2 is not meant to be scored, it does provide a method for analyzing flaws found in both critical and non-critical items: studies of ‘high-quality’ reveal no or a single non-critical weakness; studies of ‘moderate-quality’ reveal multiple non-critical flaws but no critical flaws; studies of ‘low-quality’ reveal a single critical flaw with or without non-critical weaknesses; and studies of ‘critically low quality’ reveal multiple critical flaws with or without non-critical weaknesses (Shea et al., 2017).^1^

### Statistical analysis

Analyses were carried out using software R (R Core Team, 2020) with the packages *meta* (Balduzzi, Rücker, & Schwarzer, 2019), *metafor* (Viechtbauer, 2010), and *tidyverse* (Wickham et al., 2019). Due to the significant level of expected heterogeneity between reviews, a random-effects meta-analyses model was used. The outcomes were the mean PTSD prevalence with 95% CIs, heterogeneity, and *p*-value. Between-study heterogeneity was assessed with the *I*^2^ metric (Ioannidis, 2009). *I*^2^ has a range of 0% to 100%, and for values of 25%, 25–49%, 50–74%, and >75%; it is categorized as low, moderate, large, and very large, respectively (Green & Higgins, 2009). Funnel plot and Egger tests (Egger, Smith, Schneider, & Minder, 1997) were carried out to address potential publication bias (Sterne et al., 2011).

According to the aims of the current work, different meta-analyses were run. First, we run an overall meta-analysis including all the papers meeting the inclusion criteria and aiming at assessing the general prevalence of PTSD. Then, deviating from the registered protocol, we run two additional meta-analyses to understand whether the results could have been influenced by: (i) the inclusion of underage (we thus repeated the analysis on adults only); (ii) the inclusion of meta-analysis with low quality (we thus repeated the analysis removing meta-analysis with low or critically low quality). Second, we run two umbrella reviews on papers where PTSD was assessed using self-report or structured interviews, in order to assess the impact of the methodology applied to determine prevalence of PTSD. Finally, we run two additional umbrella reviews on meta-analysis intentional and not intentional stressful events, in order to assess the impact of the nature of the stressful event on PTSD prevalence.

To directly compare the results of two meta-analyses (e.g., intentional *v*. not intentional stressful events), independent sample *t* tests were applied.

## Results

The systematic search yielded 106 records. After duplicate removal and title and abstract screening, 77 full-text articles were retrieved. Out of them, 59 articles (including 65 meta-analyses, as one article [Sepahvand et al., 2019] consists of six studies, one for each type of traumatic event) met the inclusion criteria for umbrella review (Fig. 1).

### Characteristics of the included meta-analyses

The meta-analyses included in this umbrella review had examined the prevalence of PTSD in different populations (adults *n* = 41, adolescents and children *n* = 6, heterogeneous samples *n* = 18) from different countries who have experienced multiple kinds of traumatic events, such as sexual violence (*n* = 1), natural disasters (*n* = 10), road traffic accidents (*n* = 4), illnesses that were either their own or of their loved ones (*n* = 16), circumstances related to armed conflicts and terrorist attacks (*n* = 13), immigration status (*n* = 6), incarceration (*n* = 2), murder (*n* = 1), etc. Thirty (46%) meta-analyses considered traumatic events of an intentional nature, 27 (42%) examined non-intentional trauma, and the final 8 (12%) examined the prevalence of PTSD in situations where the precise nature of the traumatic event could not be determined. Table 1 shows the characteristics of the 65 meta-analyses included in the present umbrella review.

All included meta-analyses, except for six (Sepahvand et al., 2019, *n* = 133; Sepahvand et al., 2019, *n* = 200; Sepahvand et al., 2019, *n* = 681; Badenes-Ribera, Molla-Esparza, Longobardi, Sánchez-Meca, & Fabris, 2021, *n* = 754; Al-Saadi, Chan, & Al-Azri, 2022, *n* = 755; Sepahvand et al., 2019, *n* = 779), included >1000 cases, ranging from 1093 to 494 589. Of the 65 meta-analyses considered, 33 (51%) included studies with a cross-sectional research design, whereas 32 (49%) of them reported both longitudinal and cross-sectional studies. Regarding the methodologies used to evaluate PTSD, 53 (82%) of the meta-analyses included studies that used both clinical interviews and self-reports, 7 (11%) reported studies that used only interviews, 4 (6%) included studies that employed only self-report methods, and 1 (1%) did not specify the type of assessment. Furthermore, of the 65 meta-analyses, 25 (38.5%) were of high quality according to the AMSTAR-2 scoring system, 21 (32.3%) were of moderate quality, 10 (15.4%) received a low-quality rating, and 9 (13.8%) were considered of critically low quality (see Table 1).

### Overall prevalence of PTSD

**Figure 2.**
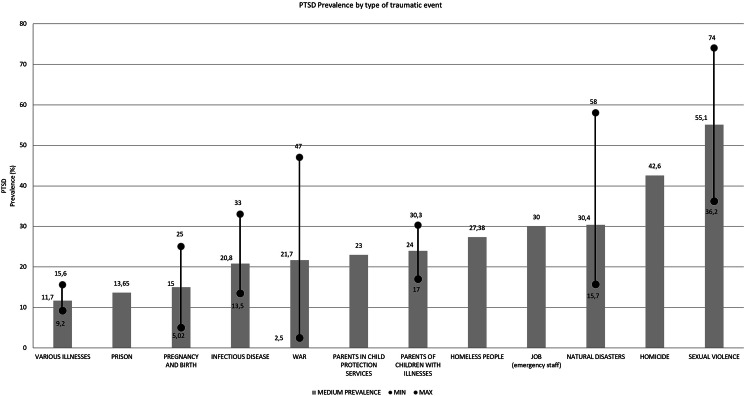
PTSD prevalence by type of traumatic event.

The overall prevalence of PTSD ranges from a low of 2.5% (95% CI 1.6–3.4) in a study on service personnel in conflict zones (Rona et al., 2016), to a high of 74% (95% CI 67–80) in a paper aimed at investigating the prevalence of PTSD following sexual violence (Sepahvand et al., 2019). Figure 2 depicts the distribution of PTSD prevalence by the category of traumatic event. The studies included in the different meta-analyses were generally found to have high levels of heterogeneity ranging from 59.2% (Aromataris et al., 2015) to 92.64% (Cénat et al., 2021); the only exception was the study by Badenes-Ribera et al. (2021) on the proportion of PTSD diagnoses following the commission of homicide (42.6%; 95% CI 38.0–47.4; *I*^2^ = 19.5%).

**Figure 3.**
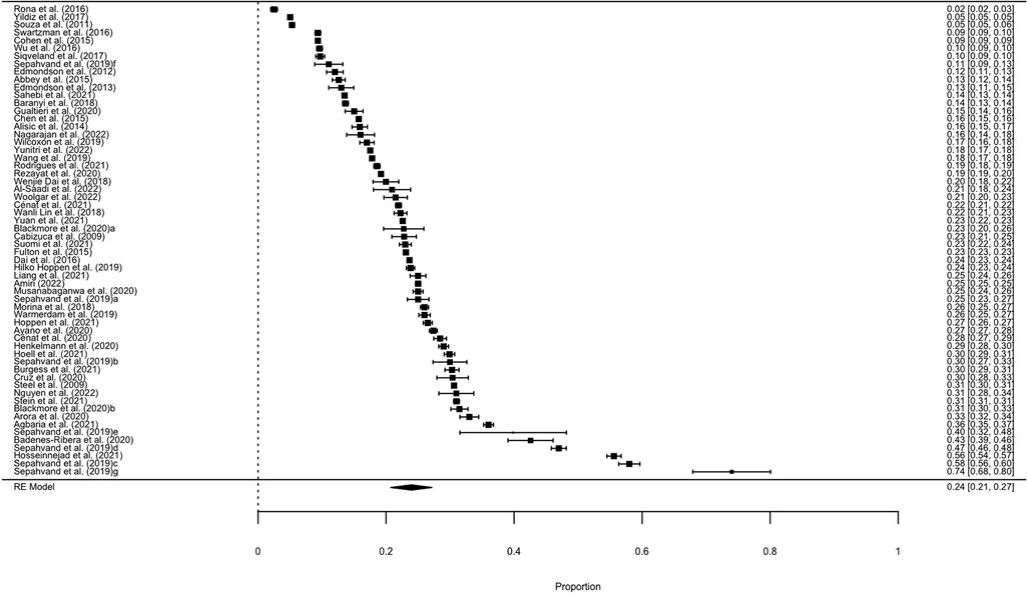
Forest plot with the outcome of the umbrella review on PTSD prevalence.

Based on the random-effects meta-analysis model, the overall prevalence of PTSD was estimated to be 23.95% (95% CI 20.74–27.15; *p* < 0.0001; *I*^2^ = 99.98%; s.e. = 0.02). Five meta-analyses that lacked information on sample size and the number of PTSD diagnoses were ineligible for inclusion in the analysis (DiMaggio & Galea, 2006; Dworkin, 2020; Hines, Sundin, Rona, Wessely, & Fear, 2014; Loignon, Ouellet, & Belleville, 2020; Van Praag, Cnossen, Polinder, Wilson, & Maas, 2019). The forest plot (see Fig. 3) illustrates both the PTSD prevalence from each meta-analysis and the overall prevalence. There was no evidence of publication bias or significant small-study effects, as suggested by the visual inspection of the funnel plot (see online Supplementary material S2) and by the Egger test, which was not statistically significant (*p* = 0.19).

Repeating the analysis on studies including an adult only sample did not change the results, with a total prevalence of PTSD of 23.03% (95% CI 18.58–27.48, *p* < 0.0001; *I*^2^ = 99.98%; s.e. = 0.02). The meta-meta-analysis carried out only on studies with high quality yielded a prevalence of PTSD of 24.26% (95% CI 20.46–28.06, *p* < 0.0001; *I*^2^ = 99.97%; s.e. = 0.04), while the analysis performed on studies with low or critically low quality resulted in a PTSD prevalence of 23.16% (95% CI 17.02–29.30, *p* < 0.0001; *I*^2^ = 99.98%; s.e. = 0.06). A two-sample *t* test was performed, and these results proved not to be statistically significantly different (*p* = 0.75).

### Prevalence of PTSD using structured clinical interviews *v*. self-report measures

**Figure 4.**
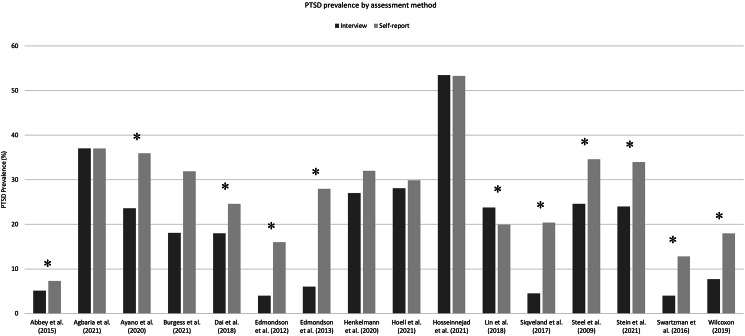
PTSD prevalence by assessment method.

To clarify whether the PTSD prevalence depends on the method of assessment used, a comparison of 16 meta-analyses that included both studies using structured clinical interviews and studies employing self-report instruments for the evaluation of PTSD following the same traumatic experience was conducted. The results are displayed in Fig. 4. In 13 out of 16 meta-analyses, the use of structured clinical interview led to lower PTSD prevalence than the use of self-report instruments (Abbey, Thompson, Hickish, & Heathcote, 2015; Ayano, Belete, Duko, Tsegay, & Dachew, 2021; Burgess, Wilcoxon, Rushworth, & Meiser-Stedman, 2021; Dai et al., 2018; Edmondson et al., 2012, 2013; Henkelmann et al., 2020; Hoell et al., 2021; Siqveland, Hussain, Lindstrøm, Ruud, & Hauff, 2017; Steel et al., 2009; Stein et al., 2013; Swartzman, Booth, Munro, & Sani, 2017; Wilcoxon, 2019), and this difference was found to be statistically significant in nine studies (Ayano et al., 2021; Dai et al., 2018; Edmondson et al., 2012, 2013; Siqveland et al., 2017; Steel et al., 2009; Stein et al., 2013; Swartzman et al., 2017; Wilcoxon, 2019). Regarding the remaining three meta-analyses (Agbaria et al., 2021; Hosseinnejad et al., 2022; Lin, Gong, Xia, & Dai, 2018), two found no difference in the prevalence of PTSD based on the assessment method (Agbaria et al., 2021; Hosseinnejad et al., 2022), whereas one reported a statistically significant opposite finding (Lin et al., 2018).

In addition to this qualitative comparison, we performed a two-sample *t* test to compare the prevalence of PTSD related to the use of these different assessment methods, which proved not to be statistically significantly different (*p* = 0.08).

### Prevalence of PTSD after intentional *v*. non-intentional events

The meta-meta-analysis conducted on studies evaluating intentional traumatic events (*n* = 25) revealed a PTSD prevalence of 25.42% (95% CI 19.76–31.09; *p* < 0.001; *I*^2^ = 99.99%, s.e. = 0.03). A lower PTSD prevalence 22.48% (95% CI 17.22–27.73; *p* < 0.001; *I*^2^ = 99.96%, s.e. = 0.03) was found in the analysis conducted on studies assessing non-intentional traumatic event (*n* = 24). However, this difference in the prevalence of PTSD was found not to be statistically significant (*p* = 0.46). Therefore, the results of the studies conducted by Breslau, Pietrzak, and their respective research teams (Breslau, 2009; Pietrzak et al., 2011) were not replicated.

## Discussion

The main purpose of this umbrella review was to provide an estimation of PTSD prevalence and clarify whether the prevalence changes depending on the assessment method used and the nature of the traumatic event. The overall PTSD prevalence amounted to 23.95% with a high level of heterogeneity between the meta-analyses. Variability in prevalence rates can be attributed to different factors and their interactions. The methodological differences between the meta-analyses and the studies contained in them, including small samples and sampling methods, the nature and severity of the traumatic event, the composition of the afflicted, the diagnostic method selected, the number of stressful events already experienced by individuals, and so on, might have impacted the heterogeneity of prevalence estimates. The main results of the umbrella review are not influenced by the quality of the meta-analysis included, highlighting the robustness and consistency of the results.

The results are not influenced by the kind of population (adults *v*. children) included, despite scientific community previously suggested that children and adolescents typically exhibit a lower prevalence of PTSD following exposure to traumatic events compared to adults (Cohen, Issues, & Issues, 2010; Copeland, Keeler, Angold, & Costello, 2007; McLaughlin et al., 2013; Tedeschi & Billick, 2017; Van der Kolk, 2003). However, it is essential to acknowledge that this apparent discrepancy in rates might be attributed to the lack of developmentally informed diagnostic criteria (Tedeschi & Billick, 2017). Furthermore, a growing body of evidence supports a multifactorial etiology for the development of PTSD in children, which appears to be even more complex than in adults. This comprehensive framework incorporates a combination of neurobiological, psychological, social, and genetic factors. Among the numerous components modulating the pathogenesis of PTSD in youth, three key factors have been consistently identified in multiple studies: the severity and nature of trauma exposure, levels of parental distress, and the duration of trauma exposure, sometimes acting as protective factors (Foy, Madvig, Pynoos, & Camilleri, 1996; Tedeschi & Billick, 2017).

The investigation of PTSD prevalence based on the assessment method revealed interesting findings. While on the one hand, the quantitative comparison of the prevalence of PTSD by applying different assessment methods did not yield significant results; on the other hand, from the qualitative comparison it was discovered that, overall, the use of structured clinical interview results in a lower prevalence of PTSD than the use of self-report instruments after considering 16 meta-analyses of studies that had used both clinical interviews and self-report instruments to evaluate disorder prevalence following exposure to traumatic events of the same nature. This difference was found to be statistically significant in 9 out of 16 studies. Regarding the remaining meta-analyses, two studies showed no statistical difference in terms of the choice of assessment method, whereas the last one reported the opposite result, showing a lower prevalence following the use of self-report measures. The outcome of the qualitative comparison is in agreement with previous studies, which confirm that the prevalence of psychiatric disorders is often higher when measured with self-report instruments than when clinical interviews are conducted (Edmondson et al., 2013). Indeed, although the use of questionnaire-based screening instruments is preferred by many practitioners for clinical settings due to the ease and velocity of administration, low cost, and wide availability in many languages, it is well known that there is considerable variation in sensitivity – the ability of the test to accurately recognize as positive those who present with the disorder (PTSD in this case) – and specificity – the ability of the test to correctly identify as negative those who do not present with the disorder – between diagnostic and screening instruments used to estimate the prevalence of PTSD (Ayano et al., 2021). Specifically, as questionnaires are often constructed for screening purposes, they provide cut-offs for the likely diagnosis of PTSD biased toward sensitivity rather than specificity (Siqveland et al., 2017; Terhakopian, Sinaii, Engel, Schnurr, & Hoge, 2008). This is related to the fact that, as suggested by Henkelmann et al. (2020), self-report measures only provide the caseness of a mental disorder (i.e. a screening condition qualifying for thorough clinical assessment), whereas clinical interviews provide a formal diagnosis. This supports the perspective, shared by researchers such as Swartzman et al. (2017), that self-report measures, despite potentially effective indicators of symptomatology, should be used with caution as diagnostic tools. Regarding the opposite results recorded in a study conducted by Lin's (Lin et al., 2018) research group, the discrepancy might be attributed to the different origins of the samples taken into consideration by the individual studies. In particular, the studies that had employed structured interviews were more likely to recruit participants in clinical sites with more serious injuries, whereas the studies that had employed self-report questionnaires were more likely to recruit participants in population-based sites with moderate injuries. Finally, with respect to the meta-analyses in which no difference was recorded on the prevalence of PTSD based on the selection of evaluation technique, the inconsistency of the results with those of previous similar studies could be due to the imbalance in the proportion of studies that had used clinical interviews *v.* those that had used self-report instruments.

In terms of the traumatic event's nature, the meta-meta-analysis on intentional events yielded a PTSD prevalence of 25.42%, while the prevalence of PTSD following non-intentional events was found to be slightly lower (22.48%), resulting in not statistically difference. This outcome is not in line with earlier research that demonstrated that sexual violence and other intentional traumas had more severe and incapacitating psychological effects than exposure to non-intentional traumatic events (Breslau, 2009; Pietrzak et al., 2011; Santiago et al., 2013). However, both Santiago et al.'s (2013) and North, Oliver, and Pandya's (2012) studies showed that, when controlling for the conditions prior to the traumatic events and the characteristics of the sample, the highlighted differences were no longer present. This suggests that the variation in PTSD prevalence observed when comparing intentional and non-intentional events may be primarily due to population characteristics and contextual issues (e.g., socio-economic factors, occupation, cultural differences, and available resources) and not to an actual different effect of the distinct types of traumatic events on disorder phenomenology. The lack of replication of these results might be due to the difficulty in distinguishing between the interpersonal and non-interpersonal components of specific events. For example, an individual who develops PTSD following a natural disaster may both have been in mortal danger or sustained injuries (*natural* or non-interpersonal component) as well as suffered the loss of a loved one (interpersonal component). Similarly, individuals diagnosed with PTSD because of being exposed to COVID-19 virus may have developed the disorder in response to one or a combination of several factors, such as fear for their safety, grief caused by the illness or death of a loved one, and forced isolation due to government restrictions and/or contagiousness.

This umbrella review is not free from drawbacks. First, our search was restricted to few datasets, thus some meta-analysis meeting the inclusion criteria could have been missed. Second, our main analyses include a heterogeneous sample of adults and underage individuals and, given the low number of papers presenting data on children only, a direct comparison of PTSD prevalence between adults and children was not performed. However, repeating the main analysis on previous meta-analysis including adults only, the results did not change, thus we are confident that the results here reported are reliable. Third, we did not evaluate the individual studies that were part of the meta-analyses in terms of their quality (since it fell outside the scope of the umbrella review). Fourth, the 29.2% of the meta-analyses that met our inclusion criteria fell within the low or critically low score at the quality evaluation. However, we found that excluding meta-analysis with low quality did not significantly impact the results, thus increasing our confidence on results reliability. Finally, the results obtained suffer from a very high heterogeneity, and their interpretation should thus be extremely cautious.

## Conclusion

Through this umbrella review, we have examined the prevalence of PTSD following diverse traumatic events and assessed the impact of different assessment methods, laying a strong foundation for future research, PTSD assessment, and diagnosis evaluations. Future studies on this topic should delve deeper into understanding how each predictor and risk factor influence PTSD prevalence. Novel data and methodologies that account for confounding variables are essential to comprehensively determine whether the disorder's prevalence varies based on sample age (children *v*. adults) and the type of traumatic event (intentional *v*. non-intentional).

Finally, it is vital to convert evidence-based insights into updated diagnostic guidelines widely accepted by the scientific community. Precise assessment criteria and systematic investigation protocols should be established to evaluate the disease across various contexts effectively. This concerted effort will improve our ability to diagnose and treat PTSD accurately and tailor interventions more effectively to individual needs.

## Supporting information

Schincariol et al. supplementary material 1 Schincariol et al. supplementary material

Schincariol et al. supplementary material 2 Schincariol et al. supplementary material

Schincariol et al. supplementary material 3 Schincariol et al. supplementary material

## Note

1. As suggested by the reference paper for AMSTAR-2, we adapted the checklist to fit the objectives of our study and the characteristics of the included meta-analyses. Specifically, we did not consider items 2, 3, and 10 to be decisive in the overall quality assessment due to the following rationale. **Item 2: Pre-established protocol and justification for deviations:** While it is ideal for meta-analyses to have a pre-established protocol and to justify any significant deviations, the absence of this information does not necessarily indicate a lack of quality. Many high-quality meta-analyses, particularly older ones, may not have registered a protocol in advance or clearly stated deviations. These meta-analyses can still provide valid and reliable findings based on rigorous methods and comprehensive analysis. **Item 3: Explanation of study design selection:** Explaining the selection of study designs is important for transparency, but it is less critical in a meta-analysis assessing the prevalence of a disorder. The primary concern in prevalence studies is the representativeness and methodological quality of the data rather than the specific design of the studies. As long as the included studies provide reliable and valid data on the prevalence of the disorder, the absence of an explanation for the selection criteria should not significantly impact the overall validity of the findings. **Item 10: Reporting sources of funding for included studies:** Reporting the sources of funding can help identify potential biases, but it is often not feasible due to limited reporting in the original studies themselves. Many meta-analyses may not include this information, yet still maintain high methodological standards and provide valuable insights. The absence of funding source information should not heavily influence the overall quality assessment if the meta-analysis demonstrates methodological rigor and robust statistical analysis.

## Supplementary material

The supplementary material for this article can be found at https://doi.org/10.1017/S0033291724002319.
